# Influence of Geometric Parameters of Conical Acrylic Portholes on Their Stress–Strain Behaviour

**DOI:** 10.3390/polym14051041

**Published:** 2022-03-05

**Authors:** Vladimir Kochanov, Václav Píštěk, Andrii Kondratiev, Tetyana Yuresko, Pavel Kučera

**Affiliations:** 1 Department of Design and Production of Structures from Composite Materials, Admiral Makarov National University of Shipbuilding, Heroiv Ukrainy Ave. 9, 54025 Mykolayiv, Ukraine; vykocanov@gmail.com (V.K.); tyuresko@gmail.com (T.Y.); 2 Institute of Automotive Engineering, Brno University of Technology, Technická 2896/2, 616 69 Brno, Czech Republic; kucera@fme.vutbr.cz; 3 Department of Building Technology and Construction Materials, O.M. Beketov National University of Urban Economy in Kharkiv, Marshal Bazhanov Str. 17, 61002 Kharkiv, Ukraine; andrii.kondratiev@kname.edu.ua

**Keywords:** organic glass, polymethyl methacrylate plastic, translucent element, hydrostatic load

## Abstract

Translucent elements in the form of truncated cones, which are made of organic glass, are widely used in the structures of portholes, submersible vessels, space vehicles, pressure chambers, teleboxes and other types of technical equipment. The decisive factor in designing portholes is to ensure the strength of their translucent elements. In order to reduce the weight of portholes and, accordingly, to increase the payload, it is necessary to optimise the geometric parameters of the translucent elements, which include the tapering angle and the ratio of thickness to radius of the smaller base. The paper deals with development of the applied (engineering) method for determining the stress–strain behaviour of the conical translucent elements of portholes made of organic glass under the action of a uniform hydrostatic pressure. Finite-element modelling of the translucent element of the conical porthole is performed, with the calculation of its stress–strain behaviour. External hydrostatic pressure of 10 MPa, absence of loads from the inside and continuous sliding of the translucent element with friction along the conical supporting surface of the porthole metal body are the boundary conditions for the computational model. Full-scale tests of translucent elements of portholes made of organic glass were performed under the action of uniform hydrostatic pressure. Analysis of the influence of geometric characteristics of the portholes on stress–strain behaviour showed that the increase in the tapering angle at the constant relative thickness of the translucent element reduced its axial displacement in all cases. Equivalent stresses acquire minimum values when the tapering angle is in the range from 75° to 105° (when the relative thickness increases, the optimal tapering angle becomes smaller). It is shown that the developed method for determination of the stress–strain behaviour of the conical translucent elements of portholes made of organic glass reflects the real picture of deformation and agrees with the results of full-scale tests. Results of the work allow us to choose the rational parameters of the translucent element for increasing the reliability of portholes through the creation of an effective distribution of stresses and strains in the translucent element, and improving its optical characteristics due to a relatively small deflection in operation.

## 1. Introduction

There are various designs of portholes adopted in current technology, which can be classified according to the shape and material of the translucent element [1,2]. Such diversity is attributed to the differences in operating conditions of the portholes: working pressure; temperature and aggressiveness of the environment; load pattern; image-registration method; and so on [3,4]. The translucent element of the porthole as a part of an optical system has a direct effect on image quality, and its strength predetermines the reliability of the technical equipment as a whole [5,6,7]. The translucent element is subject to strict requirements on the criteria of strength, tightness, deformation of optical surfaces under load and optical and thermophysical properties of the material [8,9,10].

Considering the indicators of strength, failure pattern, manufacturing and processing technology, it is more appropriate to use the polymethyl methacrylate plastic (PMMA or organic glass or Acrylic) as a material of translucent elements [11]. The characteristics of strength of translucent elements made of PMMA (the value of pressure at failure) depend primarily on the ratio of thickness *h* to diameter *D* of the porthole; and for conical and spherical elements, on the tapering angle *α* of the supporting surface.

The most fundamental recommendations for the design and choice of the structural type and geometric parameters of the acrylic portholes of different structural types are given in [12,13]. Paper [14] summarises the information about structural types of acrylic portholes but does not give any algorithms for selection of their optimal shape depending on operating conditions. Considerable attention in [15] is paid to the design of the porthole body and ways to ensure its tightness; however, no optimal engineering solutions are proposed. The authors of [16] found that at the tapering angle of *α* = 90° and relative thickness of *h*⁄*D* = 0.41 no tensile strains were observed in the porthole, but they did not assess the effect of design parameters on the porthole stress state.

Calculation of the stress–strain behaviour of a translucent element using classical methods of elasticity theory is very time-consuming in terms of implementation, since in the process of finding a solution, the mixed boundary conditions on three surfaces (two end surfaces and a supporting conical surface) should be fulfilled [17]. Furthermore, it is necessary to consider the influence of design parameters, as well as the coefficient of friction *f* on the support [18].

Regarding use of the bending theory of thin plates in the calculations of stress–strain behaviour of the portholes, there are always significant errors in the determination of displacements with slightly fewer errors than stress determination. This is because typical dimensions of the porthole translucent element (thickness *h* and minimum diameter 2*R*_0_) are comparable, and the theory of thin plates can be applied at *h*⁄(*R*_0_ ≤ 1⁄5) [19,20].

Currently, the stress–strain behaviour of translucent elements is often determined using numerical methods [21]. The finite-element method is the most accurate and universal algorithm for calculating the stress–strain behaviour of the class of structures under study [22,23]. The methods for calculation of conical portholes based on the finite-element method are considered in [24,25,26]. It is shown that the translucent element under the action of hydrostatic pressure slides with the friction of the conical supporting surface of the porthole body. At the same time, the elastic-plastic state of the porthole made of acrylic glass is considered in [24], but the creeping at cyclic loads is not analysed. The authors of [25] performed calculations for the portholes with *h*⁄*D* = 1, *α* = 90° and coefficient of friction on the supporting surface *f* = 0−0.2 under the action of pressure *P* = 70 MPa, which agreed well with the results of experiments [12]. However, there is no data on the applicability of this calculation method to the portholes with *h*⁄*D* ≤ 1.

Paper [27] shows that the most loaded elements of conical portholes are the edges of the low-pressure surface. For confirmation of the results, an experimental study was conducted. A few portholes were manufactured and tested under a hydrostatic pressure of 60 MPa. Paper [28] deals with the development of a “design by analysis” method and analysis of the stress state of the portholes considering the criteria of failure of PMMA to determine the pressure and area of failure. The detailed finite-element model of the conical portholes made of PMMA is developed in [29]. Based on this model, the stress–strain behaviour of the porthole is analysed, and the causes of its failure are investigated. The results show that local failure tends to occur at the corner of the porthole cone where the equivalent stresses according to the Huber–von Mises strain energy theory are maximal.

The experimental study of [30] shows the ability of PMMA to “adapt” to the shape of the porthole body. It was found that due to the plastic behaviour of PMMA, the highest mechanical stresses at dangerous points of the porthole are reduced by 64–71%. It confirmed the feasibility of use of PMMA in the optical systems’ technology, but the method to determine its reliability factor is not specified in this paper.

The experiments and numerical calculations of the stress–strain behaviour of PMMA portholes at different levels of hydrostatic pressure to develop a method of optimal designing regarding operating conditions are described in [31]. Experimental dependences of displacements of portholes made of PMMA at long-term and cyclic impacts of hydrostatic pressure are obtained.

Numerical methods are known for their versatility, but they are rather cumbersome and difficult to apply in practice [32,33]. At the same time, explicit solutions for the conical translucent elements of the portholes made of PMMA under the action of uniform hydrostatic pressure are limited by the necessity to fulfil the mixed boundary conditions and take into consideration the influence of structural parameters of a translucent element and coefficient of friction with the porthole body. These difficulties, together with the limited choice of fundamental functions from the full range of solutions for the axisymmetric problem of the elasticity theory, do not allow the boundary conditions of the translucent element to be fully met, while the most stress points are always on the surface of the deformable body. However, based on these methods it is possible to obtain relatively simple but fairly accurate applied solutions to the problems [31,34].

Based on the analysis, the current task is to develop an applied (engineering) method for the determination of the stress–strain behaviour of conical translucent elements of the portholes made of PMMA under the action of uniform pressure.

## 2. Materials and Methods

The conical translucent elements of portholes made of PMMA are studied under the action of uniform pressure. Classical methods of elasticity theory (axisymmetric problem) and the bending theory of thick plates are used to develop the applied (engineering) method for the determination of the stress–strain behaviour of the conical translucent elements. We accepted the following hypotheses: In deformation under uniform pressure the translucent element bends and slides in the porthole body; the element moves on the conical supporting surface with friction and without separation from the surface; the support reaction on the conical supporting surface decomposes at each point into normal and tangent components. To find an analytical solution, we used Love’s function from polynomials of degrees 3, 4 and 6, which were obtained using Legendre polynomials. Fulfilment of the kinematic and force boundary conditions is achieved by minimising the standard deviations of these conditions along the generating conical supporting surface. Numerical studies were conducted in the software package of the finite-element analysis ANSYS Mechanical 2019 R1. In view of the axial symmetry of the translucent-element shape and external load, a narrow sector was used as a computational model. The regular grid size was taken equal to 5 mm. For the conical supporting surface, a half-sized grid was used, which allowed us to more accurately model the translucent element’s interaction with the metal (titanium) body of the porthole. For the finite element of the porthole body, size of grid in the central zone was taken equal to 10 mm; on the supporting surface, where a sharp change in stress could be expected, 5 mm grid was used. The constructed finite-element model contained more than 10,000 Tetra10 elements (tetrahedron with 10 nodes). Size of the grid was chosen according to dimensions of the real porthole. Study of the convergence of the numerical solution showed that with this number of finite elements in the models the normal and shear stresses varied slightly (by 5% at most). Analysis of the quality of the constructed finite-element models did not reveal any critical errors. The problem of determination of the porthole stress–strain behaviour was solved in the linear setting. Full-scale tests were performed using the specialised experimental complex for hydrostatic loads of translucent elements of the portholes. Tests of portholes under the action of the hydrostatic pressure were performed in the high-pressure chamber (chamber volume was 0.06 m^3^, maximum pressure was 150 MPa). Axial displacements were measured by the mechanical dial gauge ICh-10 (Microtech, Kyiv, Ukraine) and electric-contact manometer EKM-2U (UAM, Kyiv, Ukraine) was used for pressure measurement. Pressure in the chamber was created by the pumping station UNGR–2500R (UAM, Kyiv, Ukraine). Specimens of translucent elements were made of SO-120 PMMA (Admiral Makarov National University of Shipbuilding, Mykolaiv, Ukraine) plates of 50 mm thickness by turning with subsequent polishing of the optical surfaces.

## 3. Theoretical Background

**Figure 1:**
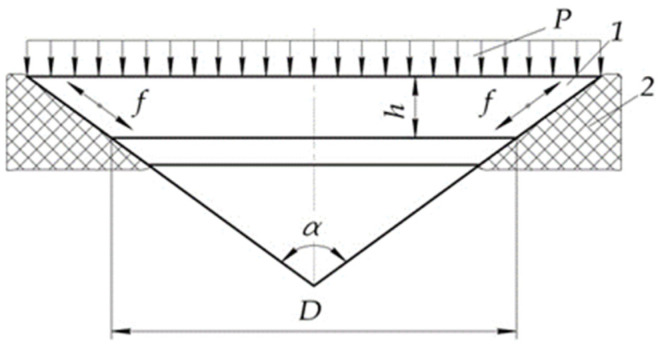
Porthole computational model: *P*—hydrostatic pressure; *f*—sliding with friction; 1—translucent element; 2—porthole body.

Material of the translucent element in the working position is under static pressure *P* from the end surface of high pressure, as well as contact (normal and tangential)-distributed loads from the conical supporting surface (Figure 1).

There are no external loads on the end surface of low pressure. Therefore, both the geometric shape of the translucent element and external surface loads are symmetric around the central axis of the porthole, so the stress–strain behaviour of the translucent element is determined by solving the axisymmetric problem of the elasticity theory.

In case of the axisymmetric problem, nonzero components of the stress–strain behaviour in the cylindrical coordinates can be expressed in terms of a biharmonic Love’s function [35,36,37] *F*(*r*,*z*):

(1) $$u=-\frac{1}{2G}\frac{\partial^{2}F}{\partial r\partial z};w=\frac{1}{2G}\left(2\left(1-\mu \right)\nabla^{2}-\frac{\partial^{2}}{\partial z^{2}}\right)F+b_{0}\;\sigma_{r}=\frac{\partial }{\partial z}\left(\mu \nabla^{2}-\frac{\partial^{2}}{\partial r^{2}}\right)F;\;\sigma_{\theta }=\frac{\partial }{\partial z}\left(\mu \nabla^{2}-\frac{1}{r}\frac{\partial }{\partial r}\right)F\;\sigma_{z}=\frac{\partial }{\partial z}\left(\left(2-\mu \right)\nabla^{2}-\frac{\partial^{2}}{\partial z^{2}}\right)F;\;\tau_{rz}=\frac{\partial }{\partial r}\left(\left(1-\mu \right)\nabla^{2}-\frac{\partial^{2}}{\partial z^{2}}\right)$$

where $\nabla^{2}=\frac{\partial^{2}}{\partial r^{2}}+\frac{1}{r}\frac{\partial }{\partial r}+\frac{\partial^{2}}{\partial z^{2}}$ is Laplace operator in the cylindrical coordinates; *u*,*w* are radial and axial displacements, respectively; *σ_r_, σ_θ_, σ_z_, τ_rz_* are radial, tangential, axial and shear stresses; *b*_0_ is free constant corresponding to the axial displacement of the body.

Biharmonic nature of Love’s function ensures the exact fulfilment of the equilibrium equations

(2) $$\frac{\partial \sigma_{r}}{\partial r}+\frac{\partial \tau_{rz}}{\partial z}+\frac{1}{r}\left(\sigma_{r}-\sigma_{\theta }\right)=0;\;\frac{\partial \tau_{rz}}{\partial r}+\frac{\partial \sigma_{z}}{\partial z}+\frac{1}{r}\tau_{rz}=0;$$

and equations of strain compatibility

(3) $$\frac{\partial }{\partial r}\left(\sigma_{\theta }-\mu \left(\sigma_{r}-\sigma_{z}\right)+\frac{1+\mu }{r}\left(\sigma_{\theta }-\sigma_{r}\right)\right)=0;\;r\frac{\partial^{2}}{\partial z^{2}}\left(\sigma_{\theta }-\mu \left(\sigma_{r}-\sigma_{\theta }\right)\right)-2\left(1+\mu \right)\frac{\partial \tau_{rz}}{\partial z}+\frac{\partial }{\partial r}\left(\sigma_{z}-\mu \left(\sigma_{r}+\sigma_{\theta }\right)\right)=0.$$

Therefore, solution to the problem of the axisymmetric stress–strain behaviour state of the body of rotation is reduced to determination of Love’s stress function. Unfortunately, at present there is no analytical representation of Love’s function satisfying any predetermined boundary conditions [35,36]. The explicit solution to biharmonic equation

(4) $$\nabla^{2}\nabla^{2}F=0$$

is possible in the cylindrical coordinates in infinite series on the Bessel functions [35,36]. However, this solution is inconvenient for practical use because of slow convergence of the series (of the order $1/\sqrt{n}$, *n*–serial number of terms of the series), particularly under difficult boundary conditions and for noncylindrical bodies [38,39]. In addition, they are inconsistent at the origin of coordinates at *r* = 0.

The solution to Equation (4) in the spherical coordinates in series using Legendre polynomials with the further transformation of the function *F* into cylindrical coordinates is relatively simple; however, it is always necessary to limit the number of terms of series in the expansion of the function *F* and satisfactory solutions are obtained for the simple boundary conditions only.

Obtaining dependences of the translucent-element stress–strain behaviour, which can be applied in practice, involves a certain compromise between accuracy and simplicity of the solution. When constructing an analytical solution, it was necessary to consider the requirements below:

- class of functions describing the stress–strain behaviour of a translucent element should satisfy the biharmonic Equation (4);
- solution should take into account the interaction of the translucent element with real support, i.e., ensure compliance with the boundary conditions thereon;
- dependences for the components of the translucent-element stress–strain behaviour should be relatively simple.

**Figure 2:**
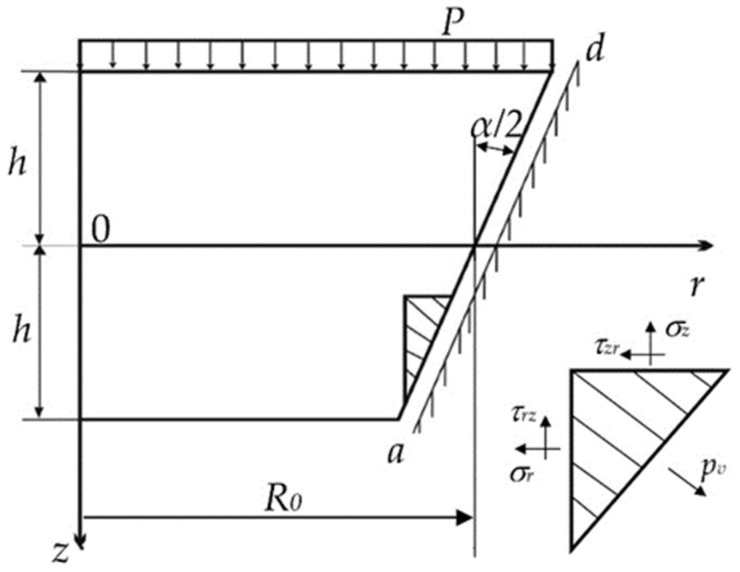
Translucent-element loading diagram: *h*—half-thickness of the translucent element; *R*_0_—average radius.

Stress–strain behaviour of the translucent element depends on its geometric characteristics, which include the tapering angle α and the ratio of the smaller base diameter to thickness *R_min_*⁄*c* (Figure 2).

When we choose the origin of coordinates in the centre of the median surface of the translucent element, boundary conditions at ends are as follows:

(5) $$\sigma_{z}\left(r;-\frac{c}{2}\right)=-p;\;\sigma_{z}\left(r;\frac{c}{2}\right)=\tau_{rz}\left(r;\frac{c}{2}\right)=0.$$

In order to fully determine the stress–strain behaviour of the translucent element, we add the conditions on the conical supporting surface *ad* (Figure 2) to the boundary conditions (5)

(6) $$P_{\tau }=fP_{n},$$

where *f* is coefficient of friction.

The following hypotheses are introduced:

- in deformation under the uniform pressure *P* the translucent element bends and slides in the porthole body along the axis *OZ*;
- the translucent element moves on the conical supporting surface *ad* (i.e., at $r=R_{0}-tg\frac{\alpha }{2}$) with friction and without separation from the surface;
- support reaction *P_v_* on the conical supporting surface *ad* decomposes in each point into normal *P_n_* and tangential *P_τ_* components.

**Figure 3:**
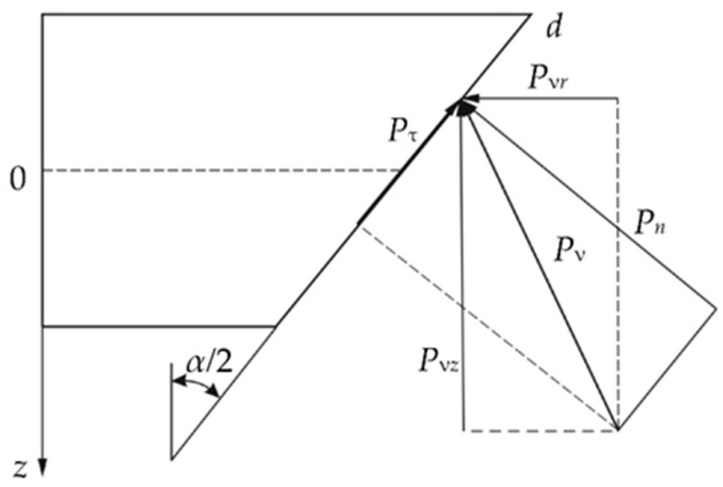
Components of the vector of support reactions.

Using the latter condition, it is possible to determine the relationship between the radial *P_vr_* and axial *P_vz_* reactions of the supporting surface. Necessary operations for this construction are shown in Figure 3.

Therefore,

(7) $$P_{vz}=P_{n}sin\frac{\alpha }{2}+P_{\tau }cos\frac{\alpha }{2}=P_{n}\left(sin\frac{\alpha }{2}+fcos\frac{\alpha }{2}\right);\;P_{vr}=P_{n}cos\frac{\alpha }{2}-P_{\tau }sin\frac{\alpha }{2}=P_{n}\left(cos\frac{\alpha }{2}-fsin\frac{\alpha }{2}\right);$$

that is,

(8) $$\frac{P_{vz}}{P_{vr}}=\frac{tg\frac{\alpha }{2}+f}{1-ftg\frac{\alpha }{2}}.$$

The translucent-element supporting conditions may be different, but they are ultimately reduced to the force boundary conditions on the conical supporting surface (i.e., the equilibrium equation of an elementary tetrahedron) and are written as:

(9) $$\sigma_{r}cos\frac{\alpha }{2}+\tau_{rz}sin\frac{\alpha }{2}+p_{vr}=0;\;\sigma_{z}sin\frac{\alpha }{2}+\tau_{rz}cos\frac{\alpha }{2}+p_{vz}=0;$$

hence, we obtain the following relationship:

(10) $$\frac{p_{vz}}{P_{vr}}=\frac{\sigma_{z}tg\frac{\alpha }{2}+\tau_{rz}}{\sigma_{r}+\tau_{rz}tg\frac{\alpha }{2}}.$$

After Equations (8) and (10), the boundary condition on the conical surface of the translucent element is

(11) $$\sigma_{r}+\tau_{rz}\beta -\frac{1-f\beta }{\beta +f}\left(\tau_{rz}+\sigma_{z}\beta \right)=0,$$

where $\beta =tg\frac{\alpha }{2}$.

Equation (11) is a condition of friction of the translucent element on the conical supporting surface. The condition of continuous sliding of the translucent element is written as

(12) u+βw=0. 

Conditions (5), (11) and (12) fully determine the surface loads and displacements of the translucent element.

Papers [35,36,38] show that for exact correspondence to the boundary conditions (5) on the end surfaces of thick plates, the degree of polynomials entering the function in *F* should not be higher than 6. To find an analytical solution, it is convenient to form Love’s function of polynomials of degrees 3, 4 and 6, obtained with the use of the corresponding Legendre polynomials [35,36]:

(13) $$F=F_{3}+F_{4}+F_{6};\;F_{3}=a_{3}\left(2z^{3}-3r^{2}z\right)+b_{3}\left(r^{2}z+z^{3}\right);\;F_{4}=a_{4}\left(8z^{4}-24r^{2}z^{2}+3r^{4}\right)+b_{4}\left(2z^{4}+r^{2}z^{2}-r^{4}\right);\;F_{6}=\frac{1}{3}a_{6}\left(16z^{6}-120z^{4}r^{2}+90z^{2}r^{4}-5r^{6}\right)+\left(b_{6}8z^{6}-16z^{4}r^{2}-21z^{2}r^{4}+3r^{6}\right);$$

where $a_{3},\;a_{4},\;a_{6},\;b_{3},\;b_{4}\;\mathrm{and}\;b_{6}$ are arbitrary constants determined during fulfilment of boundary conditions.

Substitution of (13) in (1) gives the expressions for stresses as shown below:

(14) $$\sigma_{z}=-12a_{3}+2\left(7-5\mu \right)b_{3}-192a_{4}z+8\left(8-7\mu \right)b_{4}z+320a_{6}\left(-2z^{3}+3r^{2}z\right)+\;b_{6}\left(64\left(7-11\mu \right)z^{3}-96\left(18-11\mu \right)r^{2}z\right);\;\tau_{rz}=96a_{4}r-4\left(8-7\mu \right)b_{4}z+240a_{6}\left(4rz^{2}-r^{3}\right)+b_{6}\left(-96\left(7-11\mu \right)rz^{2}+\;24\left(18-11\mu \right)r^{3}\right).$$

Fulfilment of boundary conditions of type (5) at the ends at *z* = ±*c* allows us to determine the arbitrary constants $a_{3},\;a_{4},\;a_{6}$ and *b*_6_:

(15) $$a_{3}=\frac{4\left(7-5\mu \right)b_{3}+p}{24};\;a_{4}=\frac{32\left(8-7\mu \right)hb_{4}-3p}{768c};\;a_{6}=\frac{18-11\mu }{40\cdot 704h^{3}}p;\;b_{6}=\frac{p}{4\cdot 704h^{3}}$$

After substitution of (15) in (13) we obtain Love’s function as

(16) $$F=\frac{20\left(2-\mu \right)b_{3}+p}{12}z^{3}-\frac{20\left(1-\mu \right)b_{3}+p}{8}zr^{2}+\frac{224\left(2-\mu \right)hb_{4}-3p}{96h}z^{4}+\frac{224\left(1-\mu \right)hb_{4}-3p}{32h}z^{2}r^{2}-\;\frac{224\mu hb_{4}+3p}{96h}r^{4}+\frac{p}{256h^{3}}\left(\frac{8\left(3-\mu \right)}{15}z^{6}-4\left(2-\mu \right)z^{4}r^{2}+3\left(1-\mu \right)z^{2}r^{4}+\frac{\mu }{6}r^{6}\right)$$

The corresponding functions (1) for the components of stress–strain behaviour of the translucent element are written as follows:

(17) $$2Gu=\frac{p}{4}r+5\left(1-\mu \right)b_{3}r+\left(28\left(1-\mu \right)b_{4}-\frac{3p}{8h}\right)zr+\frac{p}{32h^{3}}\left(4\left(2-\mu \right)z^{3}r-\;3\left(1-\mu \right)zr^{3}\right);\;2Gw=b_{0}-10b_{3}\mu z-\frac{p}{2}z+\frac{3p}{8h}z^{2}-14b_{4}\left(2\mu z^{2}+\left(1-\mu \right)r^{2}\right)-\frac{3p}{16h}r^{2}+\;\frac{p}{128h^{3}}\left(-8\left(1+\mu \right)z^{4}+24\mu z^{2}r^{2}+3\left(1-\mu \right)r^{4}\right);\;\sigma_{z}=-\frac{p}{2}+\frac{pz}{4h}\left(3-\frac{z^{2}}{h^{2}}\right);\;\tau_{rz}=\frac{3pr}{8h}\left(\frac{z^{2}}{h^{2}}-1\right);\;\sigma_{r}=\frac{p}{4}+5b_{3}\left(1+\mu \right)+28\left(1+\mu \right)b_{4}z-\frac{3p}{8h}z+\frac{pz}{32h^{3}}\left(4\left(2+\mu \right)z^{2}-3\left(3+\mu \right)r^{2}\right);\;\sigma_{\theta }=\frac{p}{4}+5b_{3}\left(1+\mu \right)+28\left(1+\mu \right)b_{4}z-\frac{3p}{8h}z+\frac{pz}{32h^{3}}\left(4\left(2+\mu \right)z^{2}-3\left(1+3\mu \right)r^{2}\right).$$

Expressions (17) show that the coefficient *b*_3_ determines the uniform radial compression, while *b*_4_ is a pure axisymmetric bending of the round plate. Coefficient *b*_0_ corresponds to the movement of the translucent element as a solid object in the porthole body along the central axis *z*.

It is not possible to accurately implement the force (11) and kinematic (12) boundary conditions. Therefore, we define arbitrary constants *b*_0_, *b*_3_ and *b*_4_ by minimisation of the standard deviations of these conditions along the generating conical supporting surface. The generatrix equation in the accepted coordinate system (see Figure 1) is given by:

(18) $$r=R_{0}-z\beta .$$

Considering the mixed (force and kinematic) boundary conditions (11) and (12), we can conveniently proceed to dimensionless expressions for displacements and stresses (17). To achieve this we introduce new dimensionless variables *ζ* and *ρ*:

(19) $$\zeta =\frac{z}{h};\;\rho =\frac{r}{R_{0}};\;$$

and adopt the notation

(20) $$\frac{R_{0}}{h}=\gamma .$$

Considering (19) and (20), the generatrix equation (18) is written as

(21) $$\rho =1-\frac{\beta \zeta }{\gamma }.$$

Substituting (18) into (17) and proceeding to dimensionless coordinates according to (19)−(21), we obtain the expressions for dimensionless displacements *u*,*w* and stresses *σ_z_*, *σ_r_*, *σ_θ_* and *τ_rz_* on the conical supporting surface at *μ* = 1⁄3 as below:

(22) $$\bar{u}=\frac{2Gu}{ph}=\frac{1}{4}\left(\gamma -\beta \zeta \right)+\frac{10}{3}\left(\gamma -\beta \zeta \right){\bar{b}}_{3}+56\left(\gamma -\beta \zeta \right)\zeta {\bar{b}}_{4}-\frac{3}{8}\left(\gamma -\beta \zeta \right)\zeta +\;\frac{5\left(\gamma -\beta \zeta \right)\zeta^{3}}{24}-\frac{\zeta {\left(\gamma -\beta \zeta \right)}^{3}}{16};\;\bar{w}=\frac{2Gw}{ph}={\bar{b}}_{0}-\frac{10}{3}{\bar{b}}_{3}\zeta -\frac{1}{2}\zeta -\frac{28}{3}{\bar{b}}_{4}\zeta^{2}+\frac{3}{8}\zeta^{2}-\frac{28}{3}{\left(\gamma -\beta \zeta \right)}^{2}{\bar{b}}_{4}-\frac{3}{16}{\left(\gamma -\beta \zeta \right)}^{2}-\;\frac{1}{12}\zeta^{4}+\frac{1}{16}{\left(\gamma -\beta \zeta \right)}^{2}\zeta^{2}+\frac{1}{64}{\left(\gamma -\beta \zeta \right)}^{4};\;{\bar{\sigma }}_{z}=\frac{\sigma_{z}}{p}=-\frac{1}{2}+\frac{3}{4}\zeta -\frac{1}{4}\zeta^{2};\;{\bar{\tau }}_{rz}=\frac{\tau_{rz}}{p}=\frac{3}{8}\left(\gamma -\beta \zeta \right)\left(\zeta^{2}-1\right);\;{\bar{\sigma }}_{r}=\frac{\sigma_{r}}{p}=\frac{1}{4}+\frac{20}{3}{\bar{b}}_{3}+\frac{112}{3}{\bar{b}}_{4}-\frac{3}{8}\zeta +\frac{7}{24}\zeta^{3}-\frac{5}{16}{\left(\gamma -\beta \zeta \right)}^{2}\zeta ;\;{\bar{\sigma }}_{\theta }=\frac{\sigma_{r}}{p}=\frac{1}{4}+\frac{20}{3}{\bar{b}}_{3}+\frac{112}{3}{\bar{b}}_{4}\zeta -\frac{3}{8}\zeta +\frac{7}{24}\zeta^{3}-\frac{3}{16}{\left(\gamma -\beta \zeta \right)}^{2}\zeta ;$$

where ${\bar{b}}_{0}=\frac{b_{0}}{pc};\;{\bar{b}}_{3}=\frac{b_{3}}{p};\;{\bar{b}}_{4}=\frac{hb_{4}}{p}$.

Arbitrary constant ${\bar{b}}_{3}$ is determined from the condition of static equilibrium of the translucent element along the axis *oz*. The main vector of hydrostatic-pressure forces, *P*, acting on the translucent element, is balanced by the axial components *P_vz_* of the conical supporting-surface reactions, which in turn are correlated by relationship (8) with the reactions of radial compression *P_vr_*. Considering the equilibrium of one half of the translucent element and establishing the dependence of the normal stresses *σ_r_*(*p*), being average on diametric cross section, and considering (22), we obtain

(23) $${\bar{b}}_{3}=-\frac{3}{80}\left(\frac{{\left(\gamma +\beta \right)}^{2}}{\lambda }\right)\frac{1-f\beta }{\beta +f}+1.$$

The remaining arbitrary constants *a_0_* and *b_4_* are determined from the condition of continuous sliding of the translucent element on the conical supporting surface (12), and are written as:

(24) $$\int_{-1}^{1}{\left(\bar{u}+\beta \bar{w}\right)}^{2}\partial \zeta \to min.$$

We determine the coefficients *b*_0_, *b*_3_ and *b*_4_ from the condition of satisfaction of the boundary conditions of sliding with friction on the conical supporting surface at the minimum dispersion *D*. With the use of the least-squares method in the integral form, the equation will be as follows:

(25) $$D=\int_{-1}^{1}\left({\left(u-\beta w\right)}^{2}+{\left(\sigma_{r}+\beta \tau_{rz}-\frac{1-f\beta }{f+\beta }\left(\tau_{rz}+\beta \sigma_{z}\right)\right)}^{2}\right)\partial \zeta \to min.$$

Thus, the unknown coefficients are determined from the system of equations:

(26) $$\frac{\partial D}{\partial b_{0}}=0;\;\frac{\partial D}{\partial b_{3}}=0;\;\frac{\partial D}{\partial b_{4}}=0.$$

## 4. Numerical Implementation

**Figure 4:**
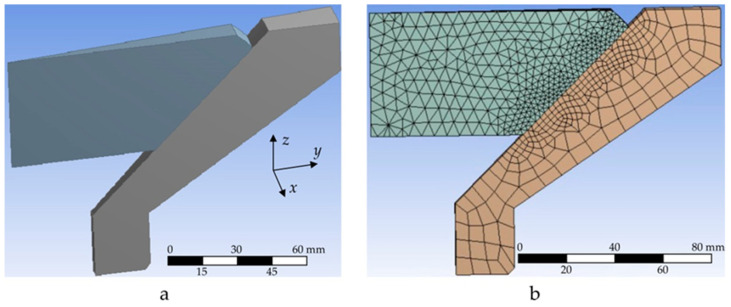
Geometric model Design Modeler (**a**) and finite-element computational model (**b**).

In view of the axial symmetry of the translucent-element shape and external load, a narrow sector was used as a computational model (Figure 4a). It allowed us to reduce the number of finite elements in the computational model of the translucent element, i.e., to reduce the time and to increase the accuracy of calculations with the same size of the finite-element grid as in the model as a whole. Since the error of the finite-element method in determination of components of the stress–strain behaviour of structures is of order $1/n^{2}$ [40,41] (*n*–order of division into elements on one coordinate), with a full-size translucent-element thickness of 48 mm, the grid size was taken equal to 5 mm. For the conical supporting surface, a half-sized grid was used; it allowed us to more accurately model the translucent element’s interaction with the metal (titanium) body of the porthole. For the finite element of the porthole body, the size of grid in the central zone was taken equal to 10 mm; on the supporting surface, where a sharp change in stress could be expected, a 5 mm grid was used. The size of the grid was chosen according to dimensions of the real porthole. The study of the convergence of the numerical solution showed that with this number of finite elements in the models, the normal and shear stresses varied slightly (by 5% at most). Analysis of the quality of the constructed finite-element models did not reveal any critical errors. The problem of determination of the stress–strain behaviour of the porthole was solved in the linear setting.

As a result, we obtained the following breakdown of the porthole structure into finite elements (Figure 4b).

Boundary conditions (5), (11) and (12) remained the same, but since a model of the narrow sector of the translucent element was used for the calculations by the finite-element method, boundary conditions of the Frictionless Support type were set on free-symmetry planes. This condition allowed us to fix the computational model on the radial sections formed by separation of a sector from the full-sized translucent element without friction (i.e., at *f* = 0) and with no normal displacements of these surfaces. The Frictionless Support condition is used on the conical supporting surface as well, at the coefficients of friction of PMMA on steel *f* = 0.2 and in absence of normal displacements of the surface. The distributed load of 10 MPa is applied to the outer-end surface; the surface of low pressure (inner-end surface) is free from external loads.

Initial data for the calculations were also elastic constants of PMMA: Normal modulus of elasticity *E* = 3000 MPa and Poisson’s ratio *μ* = 0.33.

**Figure 5:**
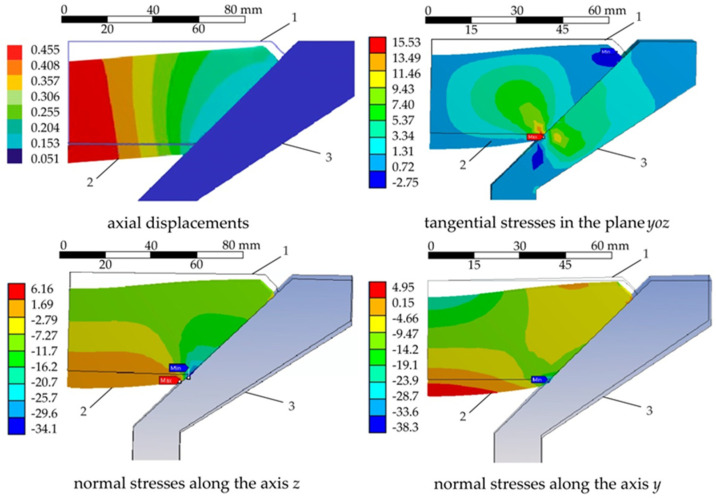
Patterns of stress–strain behaviour of the translucent element: 1—initial position; 2—after deformation; 3—porthole body.

The results of calculations of the stress–strain behaviour of the translucent element were plotted on spectral diagrams of strains and stresses (Figure 5), where each colour corresponds to a certain numerical value of the displayed indicator.

## 5. Experimental Research and Discussion of Results

**Figure 6:**
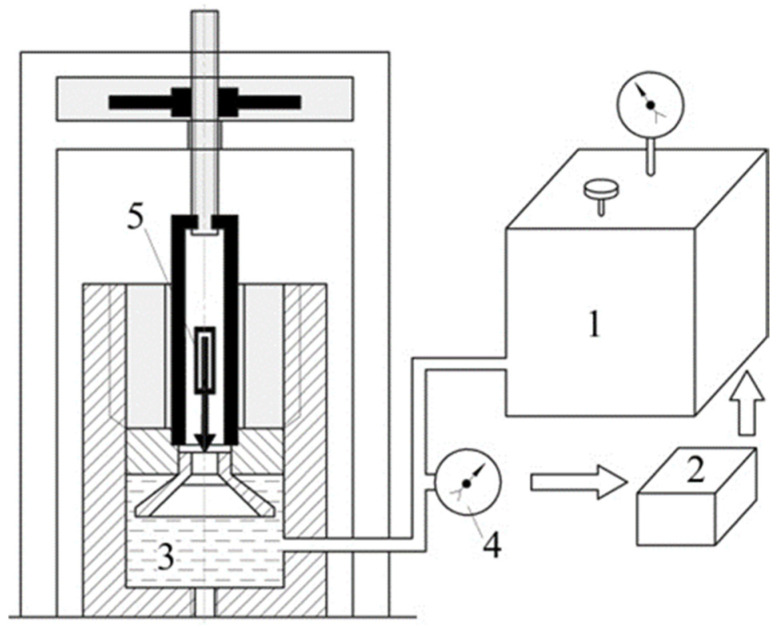
Experimental complex for porthole testing: 1—pumping station, 2—control unit, 3—high-pressure chamber, 4—electric-contact manometer, 5—electric displacement sensors.

The tests were performed on the portholes with a working immersion depth of 500 m (relative thickness *h*⁄(*D* = 0.41), tapering angle *α* = 90°) with the use of a specialised experimental complex for the hydrostatic loading of porthole translucent elements (Figure 6).

The elements of the experimental complex for porthole testing are listed below: a high-pressure chamber; a pumping station with hydrostatic pressure of up to 200 Mpa; a control unit; a stabilised electric power module; electric displacement sensors based on slide rheostats SPZ-37A; mechanical indicators of displacement of dial gauge type ICh-10; an electric-contact manometer EKM-2U; a strain amplifier “Topaz-3-01”; digital voltmeters SCH-300. The principle of operation of the experimental complex is described below. The translucent element was installed in the standard position in the lid of the high-pressure chamber. With the use of the pumping station, the working fluid (oil, water) was supplied into the high-pressure chamber and translucent elements were loaded. Pressure in the chamber was recorded by the electric-contact manometer connected to the control unit of the experimental complex.

The control unit allowed the mode of loading of translucent elements set by the operator (constant, long-term or cyclic impact of the hydrostatic pressure) to be maintained. Stress–strain behaviour of the translucent element during the test was monitored using strain gages and electric displacement sensors. A strain amplifier and digital voltmeters were used to record the results of experiments. For the automation of the process of tests and calculation of components of the stress–strain behaviour, we used the facilities of CAMAC (Computer-Automated Measurement and Control) of the standard interface. The rate of pressure rise in the working chamber was 0.6 MPa/s. During the tests, the values of pressure *P* (MPa) in the working chamber and the axial displacements of the translucent element *w* (mm) in the centre of the unloaded side were monitored.

**Figure 7:**
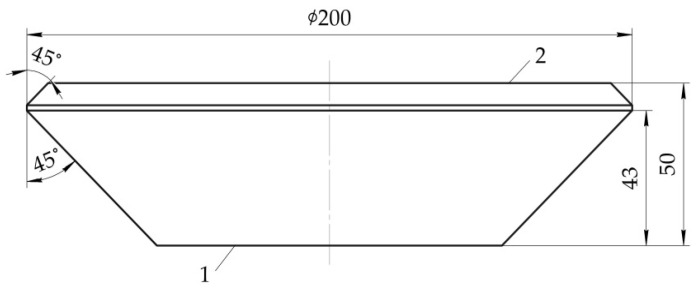
Test specimen of translucent element: 1—low-pressure side; 2—high-pressure side.

Specimens of translucent elements were made of SO-120 PMMA plates of 50 mm thickness by turning and subsequent polishing of optical surfaces. The appearance of the translucent element is shown in Figure 7.

**Figure 8:**
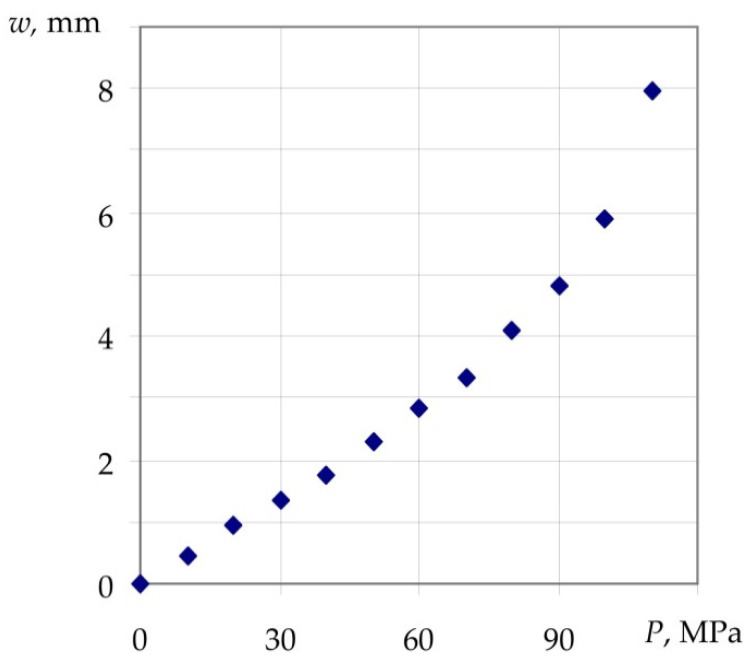
Axial displacements *w* of the translucent element at short-term loading by the hydrostatic pressure *P*.

The obtained experimental dependence of axial displacements *w* of the translucent element under short-term hydrostatic pressure *P* loading is shown in Figure 8.

Under hydrostatic loading, compressive stresses mainly occur on the surface of high pressure in the translucent element with the above geometric characteristics (Figure 8). As shown by the sharp increase in axial displacements in Figure 8, a pressure of 110 MPa can be taken as the maximum load. Dependence *w*(*P*) is linear to the level of approximately 60 MPa, which corresponds to the translucent-element axial displacement of 2.7 mm.

Table 1 shows the deflection of the translucent element under pressure of 10 MPa.

According to data of calculations using the analytical approach and finite-element method, the axial displacements of the translucent element in the centre of the low-pressure surface are equal to 0.463 and 0.455 mm, respectively. Full-scale tests show the value of deflection of the translucent element of 0.455 mm, which indicates a good agreement between the computational models and full-scale tests.

The values of movements of the translucent element at all points at the boundary of contact with the porthole body are approximately the same, i.e., the translucent element really slides along the conical supporting surface as a solid object. This phenomenon was also observed during full-scale tests of the porthole. These results confirmed the conclusions of [12,25,31].

**Figure 9:**
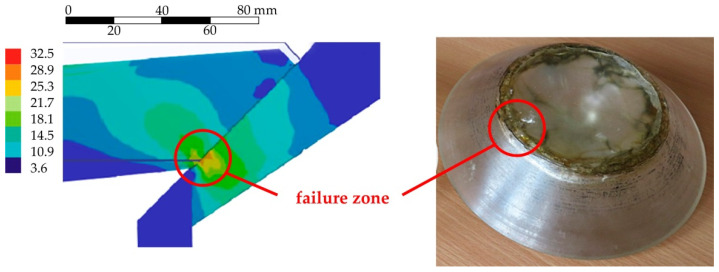
Distribution of equivalent stresses and formation of annular cracks and wrinkling area at the edge of the low-pressure surface.

Analysis of the stress–strain behaviour shows that the central zone of the translucent element (with the radius up to *R_min_*) is in a condition close to the deflection of the round, rigidly fixed plate. In the peripheral zone (with the radius of more than *R_min_*) total displacements are almost homogeneous, and the stress–strain behaviour is determined by the action of external pressure, support reactions and bending of the translucent element as a plate. The most dangerous point of the translucent element (Figure 9) is the edge of the low-pressure surface, where the equivalent stresses according to the Huber–von Mises strain-energy theory reach 32.5 MPa. According to the diagrams in Figure 6, the compression stresses are prevalent here, and they do not represent a dangerous type of stress–strain behaviour for PMMA with an ultimate tensile strength of up to 120–130 MPa [42]. After hydrostatic loading, we also observed plastic wrinkling of the material with the formation of “silver” cracks in the area of the edge of the low-pressure surface (Figure 9) of full-sized translucent elements.

**Figure 10:**
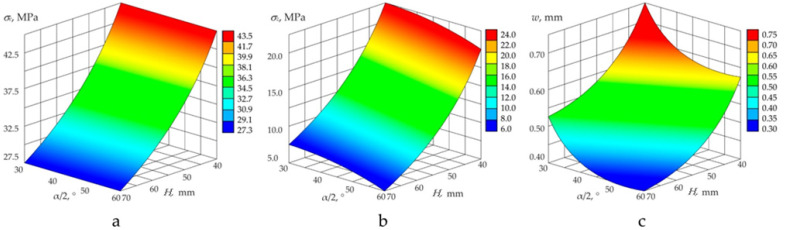
Results of calculations of stress–strain behaviour of the translucent element at the values of tapering angle varying from 60° to 150°, thickness from 40 to 70 mm, and constant diameter of the low-pressure surface (115 mm). (**a**) maximum equivalent stress; (**b**) axial stresses; (**c**) low-pressure surface.

Figure 10 shows the results of calculations of the stress–strain behaviour of the translucent element at the values of the tapering angle varying from 60° to 150°, thickness from 40 to 70 mm and constant diameter of the low-pressure surface (115 mm). Results of calculations are presented by spatial graphs of dependences of value of the maximum equivalent stress *σ_e_* (Figure 10a), axial stresses *σ_z_* (Figure 10b) at the dangerous point and displacements *w* in the centre of the low-pressure surface (Figure 10c) on the geometric parameters *α* and *H*.

Analysis of the influence of porthole geometric characteristics on the stress–strain behaviour showed that increase in the tapering angle at the constant relative thickness of the translucent element reduced its axial displacement in all cases. Equivalent stresses acquire minimum values when the tapering angle is in the range of 75–105° (when the relative thickness increases, the optimal tapering angle becomes smaller). These results confirmed the conclusions of [27,31], obtained with the use of a series of finite-element calculations.

Thus, the results indicate the adequacy of the proposed computational models and feasibility of their use to determine the stress–strain behaviour of translucent elements with the other values of geometric parameters.

## 6. Conclusions and Further Research

The paper deals with development of the new applied (engineering) method for determining the stress–strain behaviour of the conical translucent elements of portholes made of PMMA under the action of uniform hydrostatic pressure.

Finite-element modelling of the translucent element of the conical porthole was performed, with the calculation of its stress–strain behaviour.

Full-scale tests of translucent elements of portholes made of PMMA were performed under the action of uniform hydrostatic pressure.

It was shown that the developed method for determination of the stress–strain behaviour of the conical translucent elements of portholes made of PMMA reflects, in general, the real picture of deformation and agrees with the results of full-scale tests.

Results of the work allow us to choose the rational parameters of the translucent element for increasing the reliability of portholes through creation of an effective distribution of stresses and strains in the translucent element, and improving its optical characteristics due to the relatively small deflection in operation.

## Figures and Tables

**Table 1:** Deflection of the translucent element under pressure of 10 MPa.

Method of Determination	Experiment	Finite-Element Method	Analytic
computation	0.455	0.455	0.463
